# Innovative clearance data model for demining operational data collection: Insights from field trials in Zimbabwe

**DOI:** 10.1016/j.heliyon.2024.e40538

**Published:** 2024-11-26

**Authors:** R. Evans, M. Bold, L. Nelson, T. Temple

**Affiliations:** a Cranfield University, Centre for Defence Chemistry, Defence Academy of the United Kingdom, Shrivenham, SN6 7LA, UK; b Anti-Persoonsmijnen Ontmijnende Product Ontwikkeling (APOPO), Prinsstraat 13, 2000, Antwerpen, Belgium

## Abstract

From September 2023 to March 2024 a field trial on a live manual demining site was conducted to test a new and innovative approach for the collection and analysis of operational data. The approach, titled the Clearance Data Model, involved the collection of sixty-six data attributes for each mine found. For the first time the mine itself would become the accountable unit of demining data, against which an expanded range of relevant attributes particular to that specific mine would be recorded. This novel approach represented a considerable expansion to the operational data collected concerning Victim Operated Explosive Devices such as mines, booby-traps and victim operated Improvised Explosive Devices from the field. Previously few if any details about individual accountable mines were collected. The trial proved that is it entirely practical to collect such levels of data without impeding operational efficiency. It also showed that such data has significant benefits for quantitative operational risk management, as well as overall operations and quality management. For example, the recording of mine depth alongside excavation times enables oversight of individual excavation speed and management of any risk identified.

## 1 Introduction

The Clearance Data Model (CDM) represents a new way of collecting and analysing field data from the live demining sites for the purposes of operational risk management. Since the Second World War only limited amounts of data were collected about mines found during demining operations. Typically, an area would be cleared and this area, with a unique identity assigned, would have relatively few data attributes recorded about it. This minimal data would include the number of mines found in an area, the identity of the organisation that cleared the area, possibly the identity of the specific team(s) involved, requirements such as the specified depth of clearance and a summary of the procedures and equipment used [1]. This is reflected in the International Mine Action Standards Minimum Data Requirement [2]. The new CDM entails a significant change to this existing approach since it requires the collection of data attributes in relation to the individual mine rather than the area. For example, for the first time the individual mine would become the accountable unit of data. In this way more relevant and specific detail concerning the condition of the mine, its environment, how it was found and destroyed will be recorded. The approach sees the data collected on each mine not as a burden that requires more time and effort from field staff, but as an opportunity to better understand the true nature of what is being cleared and how. It also provides evidence on which semi-quantitative risk assessments can be formulated and better informed for future demining (see Table 3).

To test and verify the new CDM, from September 2023 to March 2024, it was trialled during live manual demining operations in Zimbabwe. Up to thirty-six deminers, split into six teams, recorded sixty-six data attributes for eight hundred and seventy-seven mines during the initial trial period of approximately seven months. This represents a vast expansion of the data typically collected during demining operations. This paper will describe the conduct and results of the trial. This includes adaptations made to the CDM as the trial progressed, development of standardised working practices for the field and support staff and means of establishing minimum levels of data quality.

## 2 Literature review

There is relatively little literature on the subject of data collection for mine action field operations. The main guidelines for what data is recommended to be collected are detailed in International Mine Action Standards (IMAS) 05.10, Information Management for Mine Action [3]. The standard does not identify risk management for field operations as an aim of mine action data collection, nor does it identify the individual device as the key unit of data against which attributes may be recorded. It does not list many data attributes to record for an individual device. Other publications on mine action information management, such as GICHD's Guide to Systems Engineering [4] also don't identify the need to collect detailed operational data from the field in order to assist risk management for demining and Explosive Ordnance Disposal (EOD) staff. One standard that has identified the key role that pertinent data collection can play in assisting EOD technicians is the Test and Evaluation Protocol 09.30 Explosive Ordnance Disposal and Test and Evaluation Protocol 09.31 Improvised Explosive Device Disposal.[5,6] These documents detail the competencies required of EOD technicians working in Humanitarian Mine Action (HMA) and for the first-time required staff to collect relevant data from the field for the purposes of risk management.

The secondary literature on the subject of landmines also rarely mentions the issue of data collection to inform operational decision making. Mike Croll's 2008 Landmines in War and Peace is perhaps the best overview of the subject of landmines, but while it identifies the fundamental difficulty of practically detecting mines, it does not note the lack of data on what mines have been found, in what conditions and how [7]. Ian Jones' Malice Aforethought – A History of Booby Traps [8] briefly touches on an instance of data collection but does not go to examine how the collection of operational data be more consistently applied across both military and humanitarian clearance today. Greg Lockhart's The Minefield: An Australian Tragedy in Vietnam also discusses on the collection of data about specific M-16 bounding fragmentation mines harvested for reuse from Australian minefields [9].

Recently there have been efforts to address the lack of study of how the collection of operational data can assist operational risk management. Possibly the first example was in 1945 when remarkable data was collected from the field during the demining of the Netherlands [10]. In “Know Thy Enemy and Know Yourself – The Role of Operational Data in Managing the Mines and Boobytrap Threat in Vietnam, 1965-73” the conduct of U.S Army units is examined [11]. From 1968 these units systematically collected and analysed data in a way not conducted before. This resulted in a local reduction in casualties from mines and booby traps. The crucial role that operational data plays in the land release process has also been recently identified in Land Release, A Risk Management Approach for Mine Action [12]. The theoretical Clerance Data Model was recently outlined in Operational data for the risk management of victim operated explosive devices in humanitarian mine action: A Practitioner's perspective [13].

### 2.1 The original clearance data model

The original theoretical CDM [14] was based on recording only up to fifty data attributes. The fifty data attributes were originally split into five data categories: location, environment, process, device and image. Location data for each mine would be recorded, ideally by means of a differential Global Positioning System (GPS), in order to assist risk decisions concerning what ground to clear or not to clear. Environment data was required since the immediate environment around the mine has a significant impact on the risk involved in manually clearing mines. Process data was recorded in order to better understand how demining organisations actually work. For manual demining operations this includes how the mine was found and how it was subsequently destroyed. In some respects, the gathering of process data could be deemed a form of continual time and motion study. Relevant information concerning the actual mine was captured by device data. This included detail on the specific mine model, its condition, and the potential risk this posed to the deminer. Image data was included primarily as a means of quality control, since it can be used to provide visual confirmation of the other detail recorded. The metadata of the image can also be used to verify details such as the time the mine was found, and the time it was destroyed. If a phone camera was used the general location could also potentially be verified. Table 1 shows an example of the original clearance data model. The attributes highlighted in red were not included in the revised field trial since there were not deemed necessary for the Zimbabwe manual demining context. For example, all items found in the trial minefields were destroyed in situ and therefore the distance items were moved prior to destruction was not necessary to record.

**Table 1:**
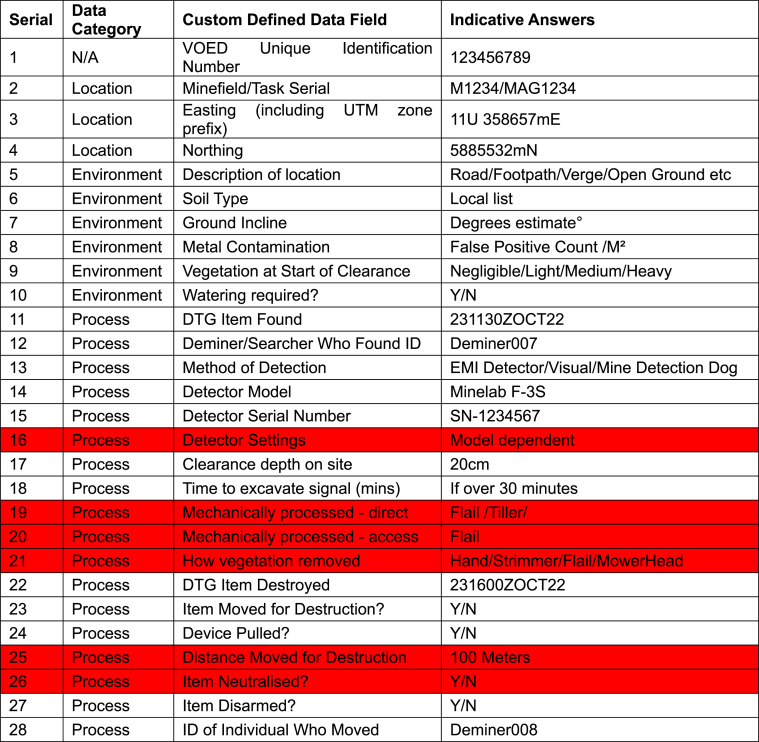

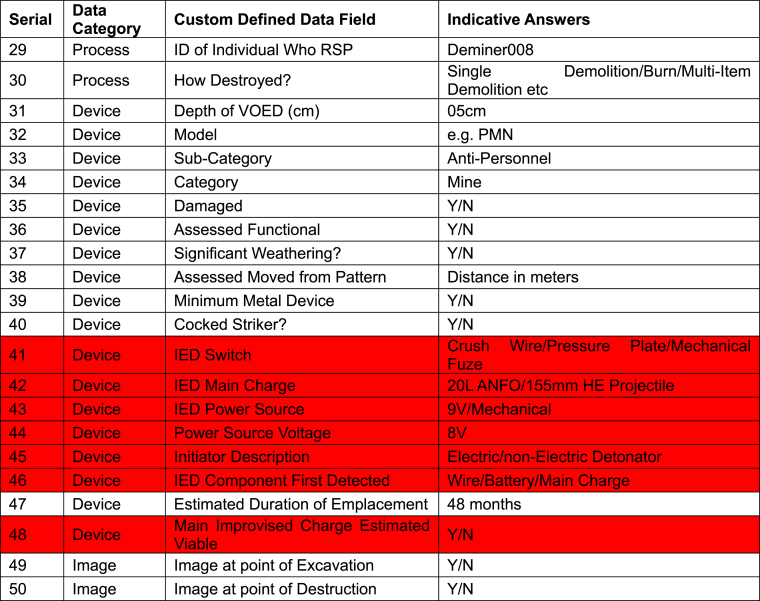
Original Clearance Data Model outlining fifty data attributes listed in the custom defined data field column. These attributes are split into location, environment, process, device and image data categories. Those attributes coloured red are those that were not included in the revised CDM for the field trial.

### 2.2 Recommended changes to The Clearance Data Model

At the beginning of the field trial the CDM was reviewed with the implementing partner Anti-Persoonsmijnen Ontmijnende Product Ontwikkeling (APOPO). As a result of this review, the original list of fifty attributes was adapted. Firstly, seven Improvised Explosive Device (IED) related data attributes were removed since the only items found in the trial location in the border minefields in Zimbabwe were conventional R2M2 anti-personnel blast mines (Fig. 5). Another 6 conventional attributes that were not relevant for the Zimbabwe field context and were also removed or changed. This was foreseen since the CDM was designed to be configurable to a given operating environment. However, another 34 attributes were added to the model during the trial, largely because APOPO requested additional process data to monitor more closely how it's deminers were working. This included process data concerned with measuring time spent on given tasks throughout the day. Deminers and their team leaders were initially required to record the overall time they expended using their Minelab detectors to search for mines, as well as the time spent removing vegetation and marking the demining lane as it progressed. In attempting to record these timings the limit of how much data it was practical to collect was found. Unfortunately, it proved unworkable for deminers to record the exact time throughout the day that they spent on vegetation cutting, using their detectors, marking, and excavating false positive signals. While exact timings proved to be too burdensome to collect, average timings were recorded alongside the specific data for each mine. In this way APOPO could achieve an overview of timings spent on specific tasks, but not achieve an exact disaggregation of deminer performance in this respect. A more deliberate time and motion study with extra resources to observe and record would be required to achieve an exact view of deminer activities. However, specific excavation and marking times for actual mines found were recorded separately and these were exact and could provide accurate data that was true to the identity of a given accountable R2M2 mine.

Other additional process data was also collected. This included the specific operational activity the deminer was engaged in when the mine was found. Within the Land Release model employed in mine action, it is typical to find mines during clearance [15]. However, it is also possible to find mines during a process called technical survey, when clearance techniques can be used in an area where there is a strong suspicion of mines, but their presence is not yet physically confirmed. In Zimbabwe this normally involved demining lanes breaching in from the border road into the suspected minefields until mine rows were found. Other activities were also included in the Drop-Down List (DDL) menu in order to assist operations managers to better understand during what land release activities mines are found. These included Explosive Ordnance Devices (EOD) spot tasks (away from minefields) and road clearance tasks. One other activity included was fade out, when demining teams clear, or at least process in some way, a margin of safety away from the cleared mine rows. Mines should ideally not be found during this process but if they are it is important that the CDM statistics reflect this since a prevalence of mines found during fade out would have risk management implications. Many mines found during fade out implies a faulty appreciation of the layout or possible spread of the mine contamination and the accompanying clearance plan.

This data is supported by the collection of another separate attribute, “moved from the pattern” yes or no. Border minefields are often traversed by the local populace for a range of reasons, and this can lead to visible mines being moved away from minefield rows or pattern. Weather events such as flooding can also move mines. It is important to measure the frequency of this to establish an evidence basis on which Land Release decision can be made. Mines found away from the pattern mean more expensive clearance. Every metre squared that is cleared represents a cost. Minimising clearance of metres squared where there is no contamination is important for operational efficiency. Mines away from the pattern are also practical to record even with basic Geographical positioning Systems (GPS) equipment. Therefore this question was included in the revised CDM implemented during the trial. However, recording a simple yes/no response to whether mines had moved within the pattern was deemed impractical, at least until differential GPS could be provided for each team.

Other attributes that were added during the course of the trial included data on weather and temperature, since the demining operator APOPO wished to monitor more closely the working conditions within which deminers operated and compare this to the process data collected. It might be assumed that higher temperatures and direct sunlight slow deminers in their clearance by making it physically harder. The capture of these attributes assembled hard data to show these effects for the first time. While cloudless days tended to be hotter for deminers with temperatures typically over 30 °C, this did not have a significant effect on the productivity of deminers. The average target excavation time throughout the working day was only 1 min more during sunny high temperature days than during cloudy lower temperature days.

One difficult attribute to measure was the metres cleared for each mine found. Historically if this was measured it would be done by a simple division of the total metres squared reported as cleared by the number of mines found. This figure would typically only be available at the end of the clearance task and would often be calculated by use of Geographic Information System (GIS) software measuring the plan view of the polygon area reported as cleared. Certain commercial operators would use Differential GPS to measure the progress of individual demining lanes throughout the clearance task. This is very accurate but also entails processing hundreds, even thousands of location points every day. This capability was not yet available at the task site during the trial period although it was ultimately intorduced in September 2024.

In this context the question of how best to record metres cleared for each identifiable mine arose, or in other words, which metres squared cleared can be fairly attributed to a given mine? The option selected was to measure and record the metres squared searched by an individual deminer since the last mine found and ascribe that to the next mine recorded. In this way the resulting dataset would be able to give at least an indication of metres squared per mine which would also serve as a useful comparison with the final overall calculations at the end of the task. It was recognised that the data collected in this way had limitations, since variation in the working patterns of individual deminers on site might skew the metres squared reported for a given mine, but the data collected still had value if those limitations were acknowledged.

Another set of process data that was added to the CDM was the explosive stores used for each demolition. This was not included in the original CDM list since often if mines can be moved, they are destroyed at a central location, either by burning or bulk demolition in order to save time (and where applicable explosive stores). However, in this instance, since the R2M2 mine was deemed a non-move item to be destroyed in situ, the mines were destroyed as individual demolitions. This meant that the explosive stores could be ascribed to an individual mine and therefore, the CDM, which is based on recording details in relation to an accountable mine rather than a period of time, or a deminer, or a team, was for this trial at least, a suitable means of explosive accounting. Since the stores used for each demolition were the same, the additional information was easy to add to each form without adding too much extra time for form filling. A manually ignited 0.9m–1m length of safety fuze initiating a non-electric detonator, in turn initiating a 0.5m length of detonation cord linking to small 37.5g pentolite charge was the standard means of destroying a single R2M2 mine. The CDM has not been designed as an explosive accounting tool but, at least in some operational circumstances, it can serve this function as well, and at least provide a check on existing explosive accounting mechanisms.

Some specific data collected for the Zimbabwe context was the presence of booster charges underneath mines. The R2M2 mine has a relatively small 60-g main charge of 1,3,5-Trinitroperhydro-1,3,5-triazine (RDX) and wax. This is enough to wound but probably not enough to be lethal. In order to increase lethality 100g 2-methyl-1,3,5-trinitrobenzene (TNT) boosters were added beneath the mines by the combatants emplacing them. The presence of these were recorded at the point of clearance. Boosters could be red, blue or green although the size was the same and there is no evidence that the colour indicated different charge sizes. The presence of a booster increased the size of an unintended detonation during excavation to 160g, although sometimes boosters were found slightly separated from the base of the R2M2 mines as the mines reorientated in the ground over time. This could possibly mean only the R2M2 mine would initiate but available accident data showed no boosters were found during post blast analysis implying these also initiated. Of the mines found during the trial, 77.3 % were found with boosters.

Orientation of mines was considered during the development of the original CDM and was included during this field trial. Mines can reorientate in the ground since the late 1970s when these were emplaced. Mines can even invert a full 180°, and 6.32 % of mines found during the trial were recorded as being found in this state. How and why this happens is not confirmed. Soil types could be a contributory factor, although of this 6.32 % both sandy and clay soils were recorded. Saturation of the ground during the rainy season that runs from October to March could be another contributory factor. Another is possibly root systems developing over time moving the 128g R2M2 mines, and where applicable the 100g boosters, within the soil. This trial was not only the first attempt to capture this data during live clearance operations, but also the first time this data has been collected in relation to other relevant factors such as soil type and topography. Soil type proved a difficult characteristic to accurately record during the trial. None of the deminers had expertise in identifying differing soil types in this area of south-east Zimbabwe. The initial options given were black soil, red soil, clay, sand, gravely and rocks but these proved unsatisfactory. Advice was sought from detector manufacturers and new options were made available in the form as the trial progressed. Visual references for each soil types were circulated amongst the staff and the soil type for each mine was reassessed by operational management staff. Where necessary soil entries were retrospectively amended using the image data.

A final extra attribute included for this particular operating environment was whether excavation was impeded by roots. The minefields cleared during this trial typically had a reasonably high degree of vegetation that had grown as the areas had been effectively fallow for over four decades. The root systems of this vegetation were significant impediments for many individual mine excavations. Where this was the case the deminer could record this with a simple yes or no answer as to whether they had been impeded in this way. Of the 877 mine excavations recorded during the trial, only 2.73 % were recorded as impeded by roots. While not part of the original CDM, roots and stones are common excavation impediments for many demining operations and therefore this question has become part of the core CDM to enable comparison with VOEDs found globally. As more data is collected any correlation between root systems and the re-orientation of the mines can be examined.

The increase in the number of attributes collected also entailed two new data categories to be added. In addition to the existing location, environment, process, device and image categories, a general information category, was added for the purpose of recording the team and the task identification code. Another new category, system identification, recorded the identifying code for each individual form and hence each individual mine, along with the date and time when the form was started. The revised CDM was therefore split into seven data categories during this field trial, as opposed to the five categories in the original theoretical model.

### 2.3 Core and discretionary data attributes

In order to prepare a wider application of the revised CDM to demining scenarios around the world, rather than just in Zimbabwe, the data attributes collected were separated into core and discretionary subgroups. Core data should be collected wherever the CDM is applied, thereby enabling comparison across all demining programmes. What data should be core and what should be discretionary was adjusted throughout the field trial. Criteria included not just data that could be collected from every operational environment, but also practicality. For example, whether an item was visually assessed as damaged was listed as core data. Assessing visually whether an item is damaged is relatively straightforward, (albeit internal damage can only be assessed by expert disassembly). However, assessing an item as functional can be more difficult, and would even more often require expert disassembly and therefore was listed as discretionary.

The core and discretion data disaggregation makes the CDM more attractive to other demining organisations since it allows flexibility to capture what data they wish given their particular operating circumstances, while also collecting comparable data for the sector. A CDM that required too many core data attributes for each mine might be less likely to be eventually more widely adopted. The onus therefore was not to include any data attribute as core unless it really would be applicable across operational environments and that operations managers would see it as relevant and worth collecting. Ultimately 38 out of 66 data attributes (57.6 %) were selected as core as a result of experience during the field trial (Table 2).

**Table 2:**
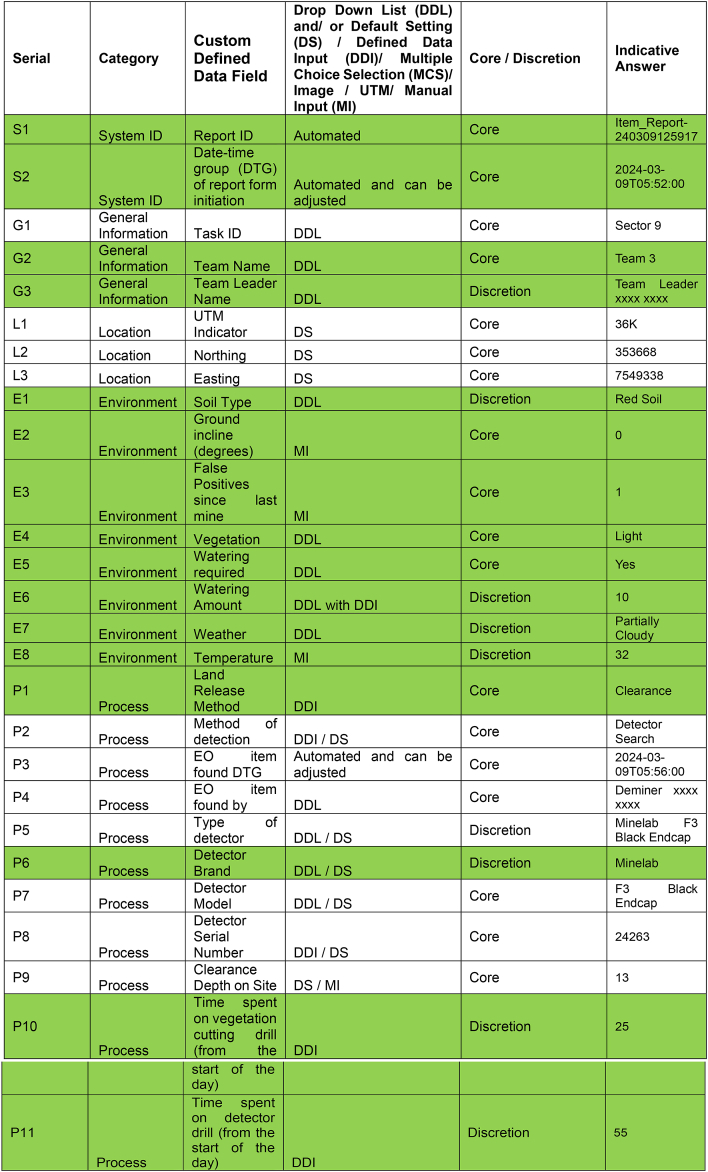

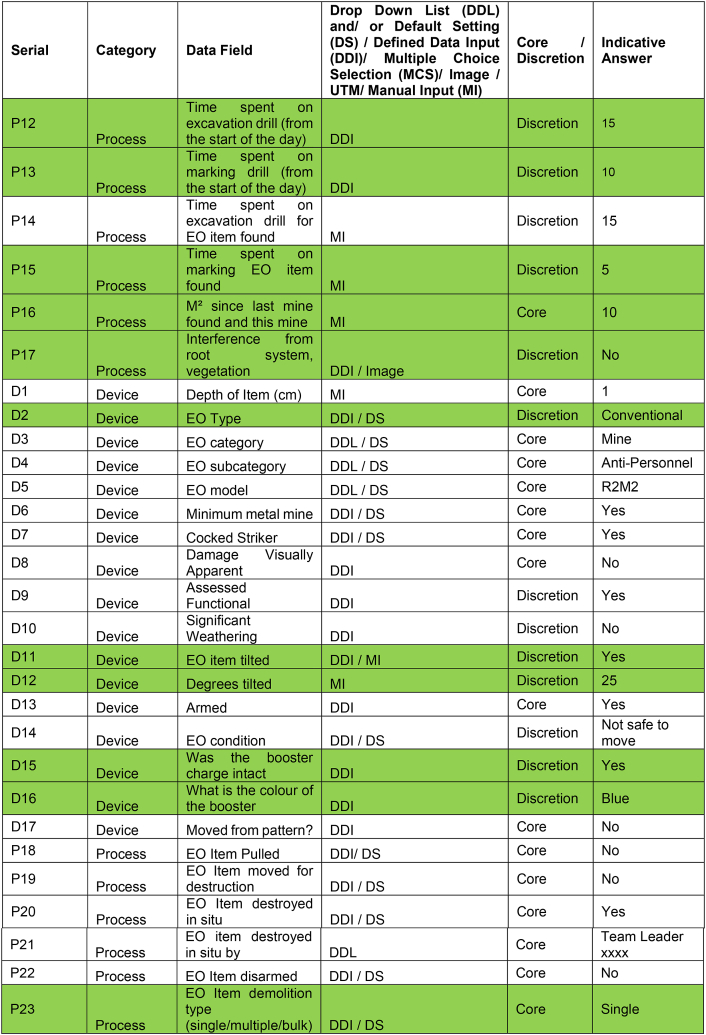

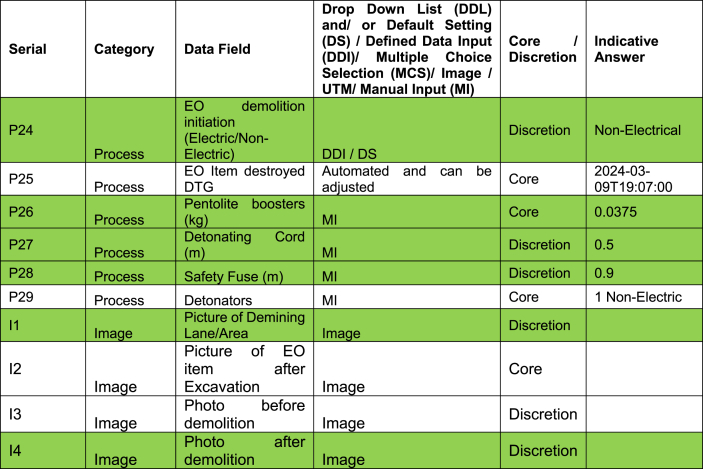
Revised Clearance Data Model showing the sixty-six data attributes collected for each of the eight-hundred and seventy-seven mines found during the field trial. New attributes developed for the field trial are highlighted in green.

## 3 Methods

The data required by the CDM during the trial was collected by means of a Specific Device Form (SDF), a new form developed for the period of the trial. The SDF was implemented using a Commercial Off the Shelf (COTS) electronic form ArcGIS Survey 123, already used within the mine action sector. The form is easy to design and subsequently adapt as changes are adopted. The initial form logic can be created using an intuitive design tool on the ArcGIS website. The SDF utilised as many custom defined fields as practical in order to maximise standardisation of data entry. The single SDFs are uploaded on a basic tablet for each demining team. The tablet has a GPS and camera function. Every time a new mine is excavated and identified by a deminer, the team leader will start a new form, the time of which is automatically recorded. The form was completed in two time periods. Of the 66 data attributes, 53 were recorded on discovery of the mine, the remaining 13 were recorded when the mine was destroyed. On demining sites, it is a common practice for mines that have been found to be left to be destroyed simultaneously, in order to minimise disruption to the actual demining. Due to lack of cellular coverage in the field, SDFs were completed off-line on site, and then uploaded every evening by 4G Wi-Fi router at the camp. The data was held on multiple ARCGIS online servers, enabling backup of data and minimising the chance of loss. The data is then held in a validation box to be checked and validated by the operations manager before being visible on the dashboard.

Each question on the SDF can be answered in one of six ways. For automated information such as the time the form is started, a Default Setting is used to automatically capture the time. Manual Input (MI) is used for numerical data that cannot be automated, such as the depth at which an AP mine is found. Drop Down Lists (DDL) are used as much as practical in order to standardise answers. There was no manual input of text, only numbers. Other fields included four separate images with the associated metadata and UTM location data that is captured by default from the GPS integrated into the tablet. After the trial more accurate Trimble DA2 differential GPS were eventually employed that enabled 30 cm accuracies for all geospatial measurements. This will enable greater accuracy in plotting individual mine positions and analysing these in relation to other mines in the pattern.

### 3.1 Standard Operating Procedures

The CDM operating method was detailed by a written Standard Operating Procedure (SOP). This has been adapted as the implementation of the CDM evolved during the trial period. The SOP has been the basis of briefing to both team leaders and deminers. All staff are bound to abide by APOPO SOPs in their contracts, so the SOP was the best way to codify the evolving practice that implemented the CDM.

### 3.2 Example of data collected for an individual Mine

**Fig. 1:**
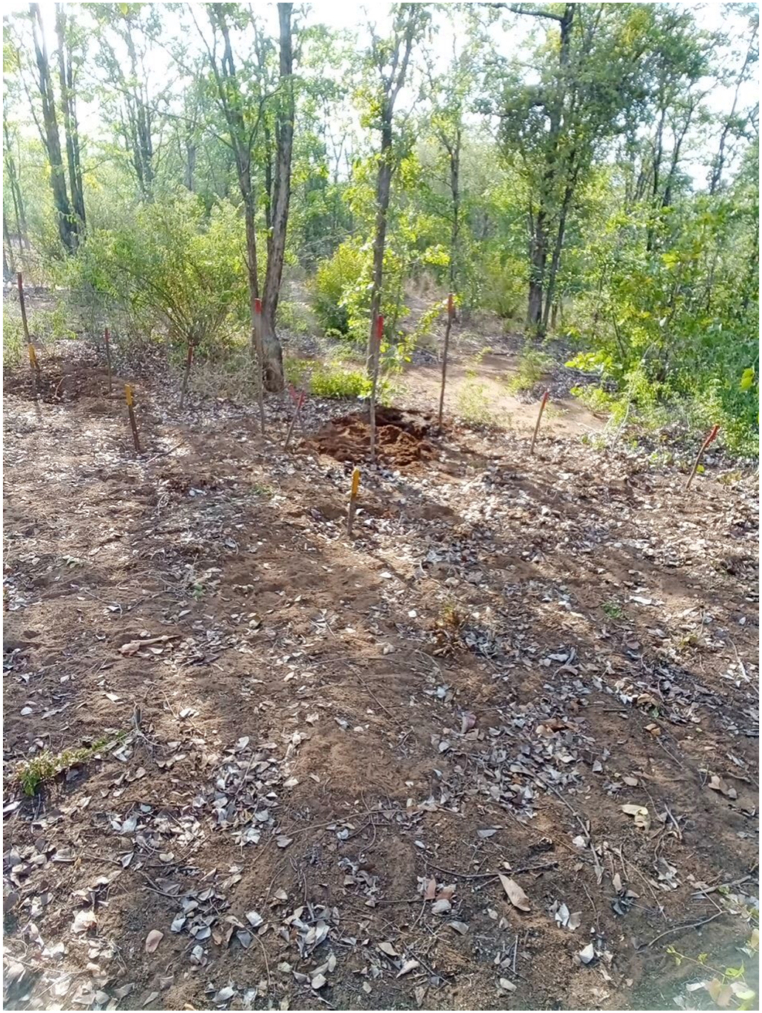
A view of the lane area where Item_Report-240309125917 was found.

**Fig. 2:**
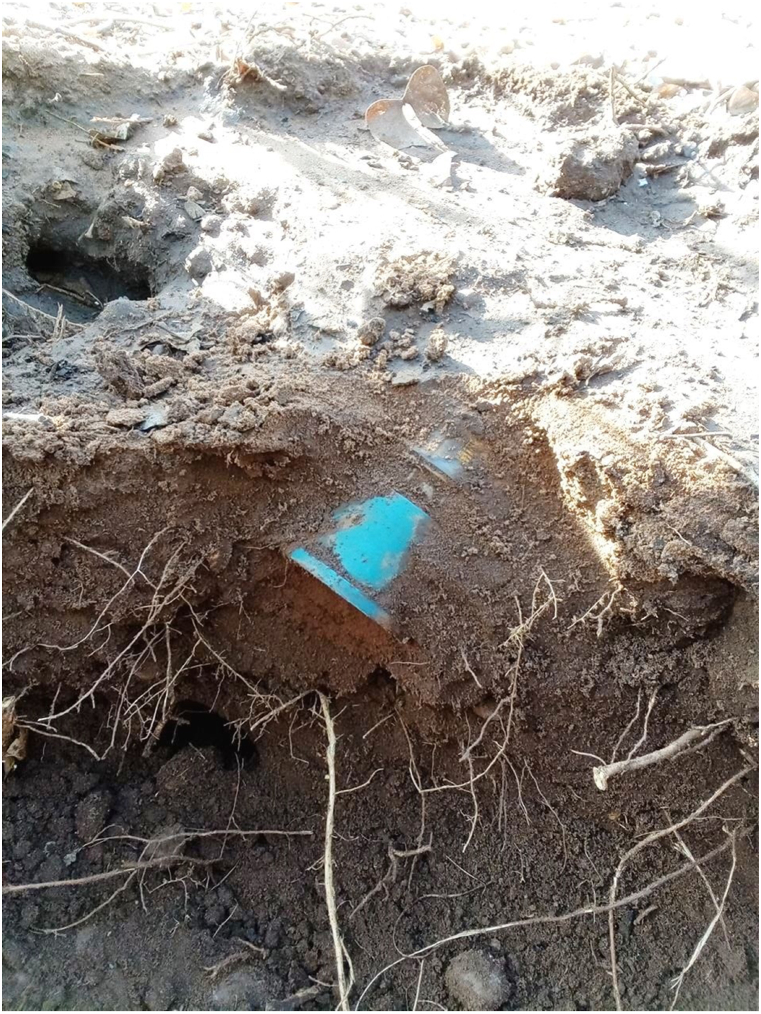
Item_Report-240309125917 of the trial photographed once excavated. The mine was estimated to have tilted 35°. The blue 100g TNT booster is clearly the visible. The deminer has excavated the mine no more than necessary to identify it and place a donor charge. (For interpretation of the references to colour in this figure legend, the reader is referred to the Web version of this article.)

**Fig. 3:**
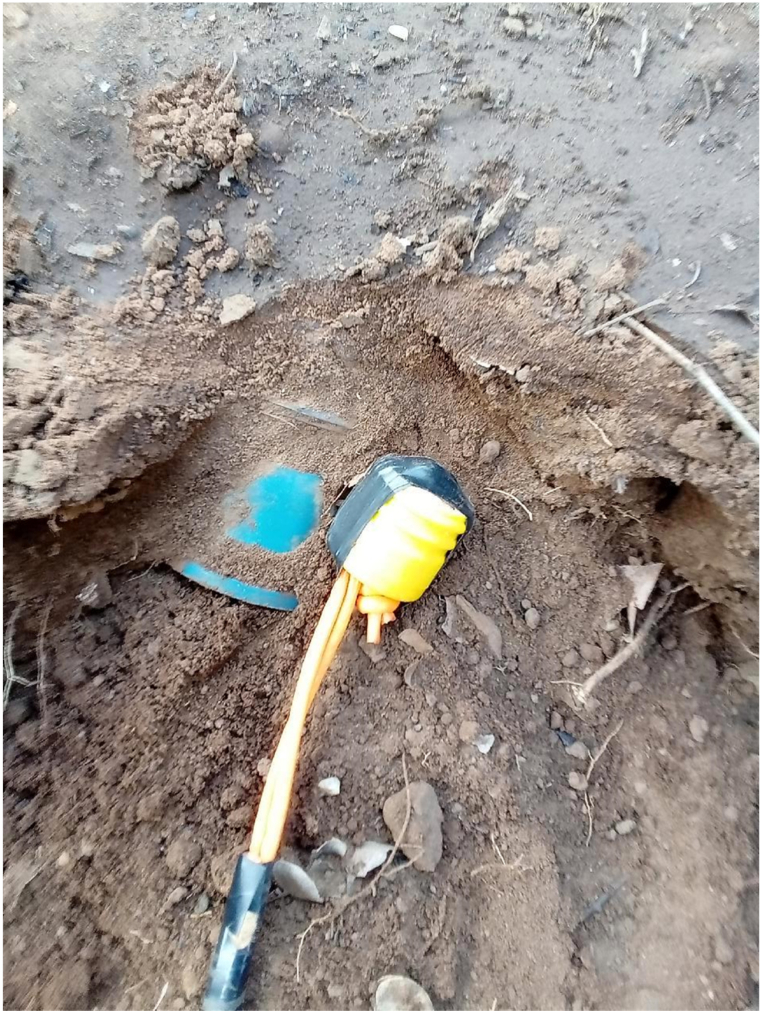
A 37.5g pentolite donor charge placed next to the mine.

**Fig. 4:**
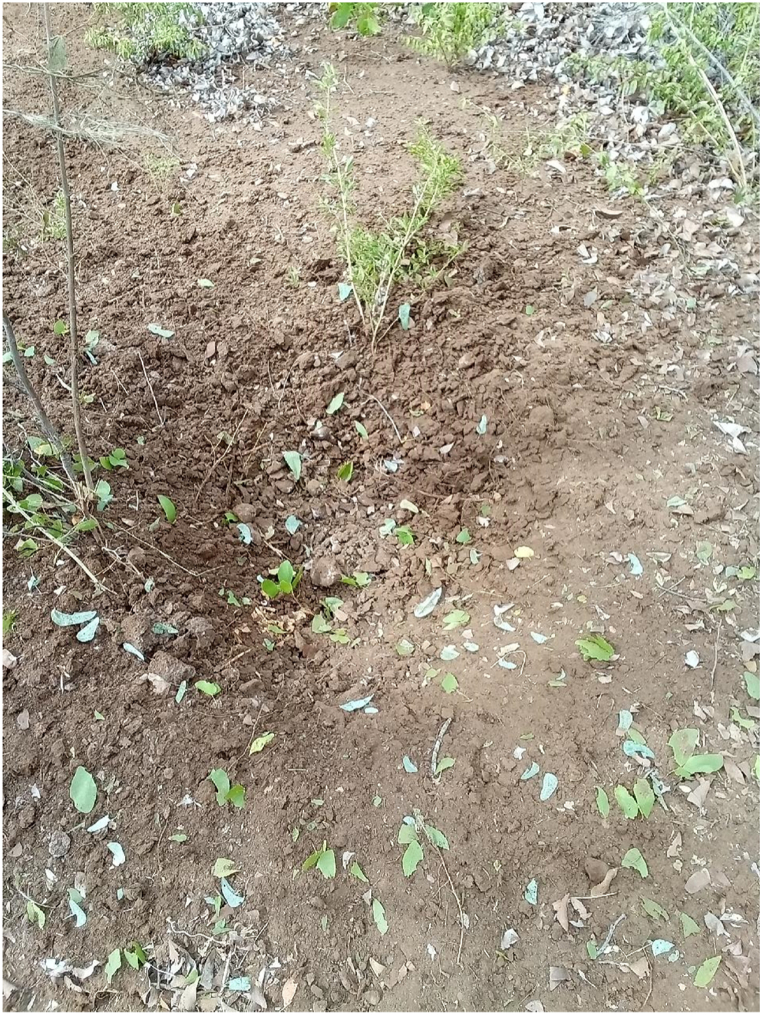
An image showing post demolition scene.

One example mine can be used to illustrate the level of detail collected during this trial. The mine was found on the morning of final day of the trial, March 09, 2024, at 06:52 a.m. The mine was given the unique identification code Item_Report-240309125917. This mine was one of seven mines found and destroyed on this date. The location data of the mine was 36K 353688 7549338. The weather was partially cloudy, the temperature was 32° centigrade. The ground was flat with light vegetation, (Fig. 1). The process data for the item showed it was detected at a depth of 1 cm (Fig. 2) by a Minelab F3 detector with a black endcap, serial number 24263. This detector, used by two deminers, found 28 individual mines during the trial period. These mines were found at depths ranging from 1 to 10 cm, with an average of 4.18 cm. The clearance depth on site was 13 cm. The deminer spent 55 min searching the lane with the detector, and 25 min cutting the light vegetation present. Target excavation of the mine itself took 15 min, even though its position was shallow. During the course of excavation 10L of water was used to soften the ground. Roots were not reported to have been an issue while excavating. No false positive signals were investigated in the demining lane prior to the target signal. The mine was destroyed in situ between 09:12 and 09:21 on the same morning using 0.9m of safety fuze, a non-electric detonator, 0.5m of detonating cord, and 37.5 g of pentolite (Fig. 3, Fig. 4). The metadata of the four images taken was used to confirm the time the mine was found and destroyed. The name of the team leader who conducted the demolition was recorded. The device itself was an R2M2 anti-personnel mine. It was found with a blue booster. Once excavated the mine was estimated to have tilted 35° from the vertical axis. From external appearance only the mine was assessed to be functional, undamaged, and not noticeably weathered.

**Fig. 5:**
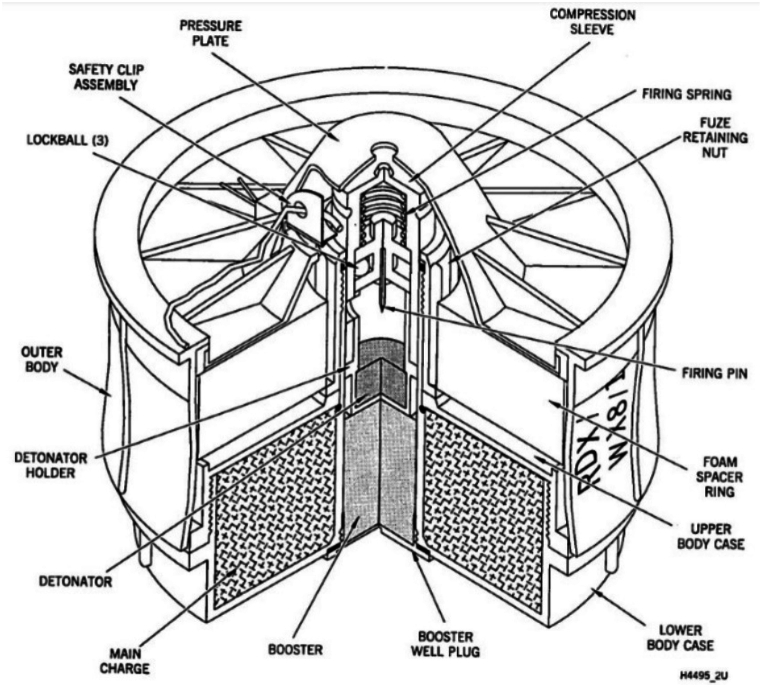
A sectioned diagram of an R2M2 anti-personnel blast mine. (Image © US DoD).

### 3.3 Analysis of data

Once validated the data could be viewed by means of a flexible dashboard. Each mine with its respective imagery and data could be scrolled through. An ability to download data in tabulated form was crucial for analysis, not least for quality control, since it enabled faster identification of anomalies and potential errors that required re-verification with the individual who completed the form. The dashboard was the depository for data that could be the basis for quantitative risk assessments. It also served as the main interface for supporting operations, enabling scrutiny of each deminer's performance but also an overview of key operational information such as average target excavation times and explosive accounting.

### 3.4 Establishing data entry quality

Data entry competence amongst operational field staff was not a codified requirement within the mine action sector until recently. In 2022 a revision of the International Mine Action Standards (IMAS) conventional EOD competencies changed this to some degree.[16,17] Now operational data collection and reporting is an obligation for EOD staff in the field operating in conformance with International Mine Action Standards. While more pertinent to EOD tasks rather than demining operations, the two disciplines overlap. This field trial saw the application of EOD Data and Reporting competencies to not only EOD demolition functions during demining tasks, but also to the strictly demining aspects as well.

An early challenge for the trial was to establish norms of data quality. Forms would be filled in by team leaders offline but on site. The new “one mine one form” approach represented a significant change for these individuals. Until the trial a simple count of mines found by a team would be listed on one daily report. Now they would be required to input sixty-six data attributes per mine. When the field trial started in September 2023 instructional briefings were held by the operations manager emphasising the need for accuracy when entering data. This was then reiterated during two follow up briefings in November 2023 and again in February 2024. Allied to this was scrutiny of the operational data collected from the field by experienced field operators who could easily identify anomalies. In addition to validation by the operations manager, data was scrutinised on at least a bi-weekly basis with specific queries fed back to the field. Where errors were confirmed for each individual team leader, corrections were made and where necessary further training conducted.

This quality control of data was conducted not only by reviewing spreadsheets of data and identifying anomalies but also corroborating against the respective image data. The CDM was originally designed to include only images of all mines found and the demolition set up for the destruction of that mine. During discussions with APOPO it was agreed that extra images of the demining lane and immediate surroundings, along with the small crater post demolition should also be taken during the trial. These images enabled an enhanced level of quality control of the data reported from the field. The data for each mine could be compared with the images for consistency. For example, the depth and orientation of each mine found were easy to check against the image of the excavated mine. The metadata of the image could also be checked to ensure it was consistent with the time and date when the mine was reported found and destroyed. The extra images of the demining lane enabled a reference to check much of the other data inputted into the form, including the levels of vegetation, the amount and quality of the marking, and the ground incline. Even details such as the watering of the ground to assist excavation could be verified by reference to the demining lane and excavation images.

The understanding that all data entry would be checked progressively shaped behaviour amongst the team leaders. Since they knew they would be accountable for the data they had entered and that this would be to a degree not previously experienced during their demining careers, greater care was taken, and the prevalence of errors decreased. Strict quality control of operational data is an ongoing task which is integral to the successful implementation of the CDM. Another means of monitoring data input that was implemented towards the end of the trial was recording the time taken to input data into the form. The form would automatically calculate the combined time from the two occasions it was opened, until it was closed. This allowed managers to not only monitor whether the form was being filled in as mines were found and destroyed, but it also enabled monitoring of the data input burden placed on team leaders. One of the possible reasons why these levels of data collection had not been attempted before was the perception that the effort would be too burdensome and could interfere with the actual demining. This time data showed that the data collection was entirely practicable, with an average form taking a combined 5–7 min to fill, and on average each team leader filling in no more than seven forms a day on the busiest days, and often far fewer. None of the deminers were disturbed in their work and productivity was not affected by collecting CDM data. The daily observed average of 25–30 m^2^ cleared per deminer was maintained. Indeed, for the first time the efficiency of each deminer was measured through the collection of process data in a way not attempted before.

Even with the constant effort expended on trying to improve the data quality, as is common with many data sets, a degree of data cleaning was required from those supervising the trial. Gross errors were often easy to spot and if necessary, these could be retroactively amended by the information management officer. For example, one input of 2121 false positives for a mine found on October 14, 2023 was straightforward to identify and address. Once checked with the team leader who filled out the entry and the deminer the figure was confirmed as a false data entry made in error and discounted. A reading of zero false positives was confirmed and entered for that mine. Other data cleaning included correcting overlength detector serial numbers which could only be five digits long. Since the serials of all detectors used by each individual deminer were known it was easy to correct and clean this data. Each instance when data was amended a record was kept in the data base in order to maintain accountability for the change.

## 4 Results and discussion

The field trial of the Clearance Data Model saw 66 data attributes collected on 877 anti-personnel blast mines. This represents the most detailed data set collected on AP mines found in the field. While the data collected had multiple uses, including for general operations management, the primary aim was to improve risk management by collecting data to enable quantitative risk assessments. The CDM data was collected with the express purpose of enabling improved risk calculations.

### 4.1 CDM based risk assessment for target Investigation of an R2M2 Mine - risk assessment

In recent years demining operators in Zimbabwe have experienced a series of accidents during the excavation of R2M2 mines. In 2021 there were four recorded, in 2022 there were three and in 2023 there were three.[18,19] The accidents resulted in injuries to the excavating hand and arm. All the deminers were wearing visors and aramid aprons during excavation so the severity of injuries was mitigated [20]. The R2M2 is a dangerous mine that is normally excavated no more than necessary. Ideally the final stages of excavation are conducted by means of a paint brush gently moving the last soil impeding visual identification. Excavation is conducted sufficiently for a donor charge to be placed as near to the body of the mine without touching it, and no more. Typically the mine will remain stabilised in the wall of the excavation trench with just one side of it revealed.

The fuzing mechanism of the R2M2 integrates a compressed spring that propels a striker to a stab detonator beneath. This is often referred to as a cocked striker. The spring and striker assembly is held in place by three locking balls acting as a holding device that is overpowered when sufficient force is applied to the pressure plate and compression sleeve above it. Any impact on or movement of the mine could be adequate to overcome the locking balls holding device, especially if the mine is damaged and the locking balls are not fully held in place by the plastic casing of the mine. Even new mines removed from the packaging only require a force of 3–7 kg to overcome the pressure plate and locking balls [21].

The data collected during the CDM trial provided a new means to better risk assess the excavation of R2M2 mines. The risk assessment format adopted (Table 3) consists of the usual severity multiplied by probability calculation, each on a scale of 1–5. However, the difference for this operational risk assessment is an additional sum that relies on CDM data. Each relevant data attribute that scores adds one point to the multiplication result. Other relevant data attributes are listed for context or explanation but don't affect the risk calculation.

**Table 3:**
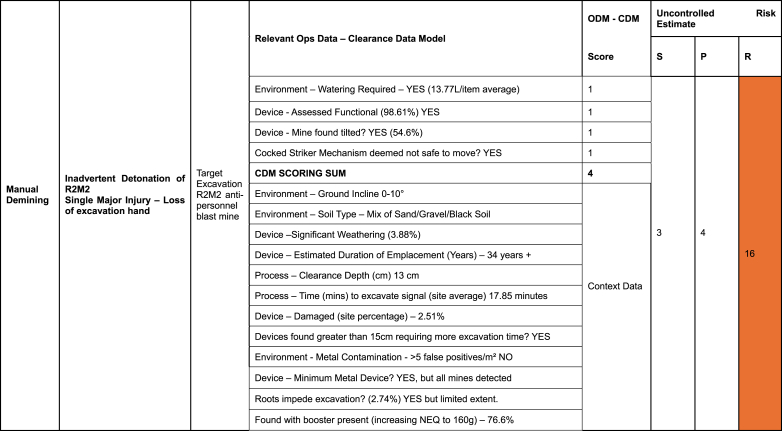

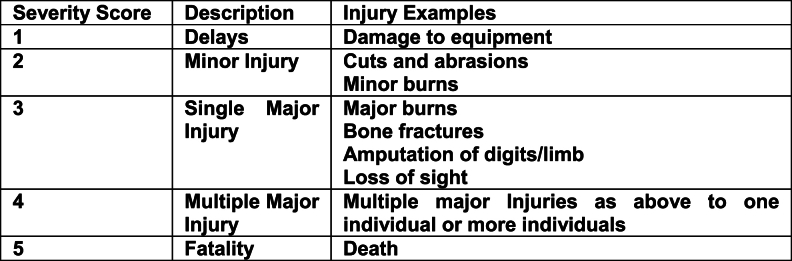

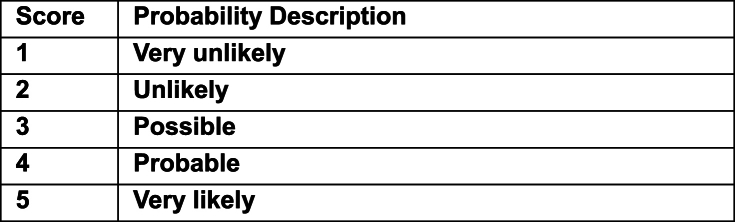

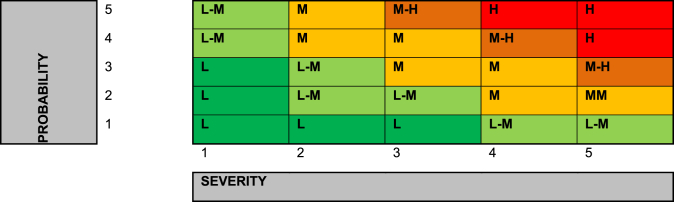
– Risk Assessment based on Clearance Data Model for Target Excavation for R2M2 Zimbabwe

Risk Score thresholds.

Low = 1–3 Low-Medium = 4–6 Medium = 7–12 Medium-High = 13–19 High = 20 - 25.

RISK SCORE = (SEVERITY x PROBABILITY) + DIRECTLY APPLICABLE OPS DATA SUM.

The key decision when populating the risk assessment was deciding what operational factors are scoring for the risk assessment calculation, and what qualified as relevant context, as the quantity of scoring risk factors affects the overall risk assessment score. Therefore, a relevant attribute had to be true for at least 20 % of the 877 mines in the sample to count as scoring, otherwise it would be relegated to the context list. For example, the presence of root systems could credibly be a scoring attribute. However, in this sample roots only affected 2.74 % of mine excavations and therefore did not affect enough excavations to score. Another example would be that R2M2s are minimum metal mines which could affect the ability to reconfirm signals during excavation. However, in this sample 98.86 % were found at depths up to 30 cm with the Minelab F3 detectors using black endcaps (high sensitivity). (The other 1.14 % of mines were identified visually since they protruded from the surface.) The relative lack of false positives due to minimal metal clutter and the presence of the mines in a pattern also assisted this. Ultimately while deminers had to move cautiously, clearing approximately 25 m squared a day, the circumstances in this location meant that mines were relatively easy to locate. Therefore, the minimum metal nature of the R2M2 was listed as relevant context rather than as a scoring attribute.

Damage or weathering could qualify as a scoring attribute but in this case only 3.88 % were assessed as weathered and only 2.51 % were assessed as damaged. These percentages were too low to qualify as scoring data. Ground incline can make excavations on steep slopes potentially more hazardous for deminers but in this sample the ground was flat with no incline more than 10°. Mines that are found deeper than the standard excavation depth of 15 cm (2 cm more than the site clearance depth) might also pose an excavation risk due to the increased excavation required but this was true of only 1.37 % of mines. Therefore, mines deeper than the excavation depth did not qualify as scoring data.

Ultimately only four data attributes were selected as scoring data. The first was the key attribute of the cocked striker fuzing system within the R2M2 mine. The accident record over the previous three years shows that R2M2 mines found were prone to initiation during excavation. 100 % of items found were R2M2 mines. The second attribute selected was the functionality of the mines. While this represents a visual assessment of the mine and not a full testing associated with technical disassembly, 98.61 % were assessed as functional by the site supervisors. Whether soil required water to assist excavation was selected since this applied to 80.27 % of excavated mines. Hard soil can induce deminers to exert too much force during excavation, thereby increasing the risk of an inadvertent detonation. Of the 704 target excavations that required water, 13.77 L were on average required. The trial was largely conducted during the rainy season that runs from October to March (the trial period was September 2023 to March 2024). The final scoring data selected was whether the mine was tilted. This applied to 54.6 % of the mines excavated. Tilted mines could potentially pose a greater risk to deminers since the sideways excavation motion is more likely to apply pressure to the base or head of the mine, and therefore the pressure plate, if that mine is titled off its vertical axis.

The risk assessment calculation assessed the results of previous excavation accidents in Zimbabwe. Injuries consistently involved traumatic damage to the excavating hand and arm. Loss of fingers was common. This would qualify as a single major injury and therefore be a score of 3 on the severity scale. Given the relative frequency of excavation accidents, a probability score of 4 was awarded, indicating that such accidents were probable. This resulted in an initial score of 12. The additional score of 4, (from the four data attributes chosen as scoring data), then made an overall risk assessment score of 16, indicating a medium high risk, which was deemed to be acceptable. Nevertheless, a review was conducted of the mitigation applied to assess whether it was sufficient to moderate the risk to a tolerable level. Was the demining organisation doing everything necessary to reduce risk to a level As Low As Reasonably Practicable (ALARP)? Deminers either wear the standard 5 mm polycarbonate visors, or Rofi protective masks made from 5 mm polypropylene with 5 mm polycarbonate eye protection. Visors, even when well cared for, become scratched and are therefore procured with a peelable protective layer to prolong service life and are changed annually. Over the front of the torso and upper leg standard para-kevlar vests are worn. The demining organisation also provided deminers with kevlar gloves in a further attempt to reduce the severity of injury although some question the efficacy of these [22]. Another risk mitigation that is being considered is the use of two-handed excavation tools that keeps each excavating hand 30 cm from any detonation. This is potentially enough to save the deminers losing fingers or their hands [23]. The two-handed tools are made from malleable stainless steel and have been trialled in manual demining operations in Lebanon. The adoption of these tools will be reconsidered in light of this risk assessment.

## 5 Limitations

While the field trial was the first time such significant volumes of data have been collected about individual mines in a live demining environment, there were certain limitations. This was a field trial in one operational environment with one anti-personnel mine model. In future it is hoped that the core attributes of the CDM will be applied in other operational contexts, and for a range of contamination including other types of anti-personnel mine and victim operated IEDS. If each processed mine recorded using the CDM is viewed as a repeatable experiment, the experiment needs to be applied to other models of mine in different conditions. The real test will be if the core data attributes, to be recorded for all mines in all conditions, are practical and that any potential global dataset enables overall risk assessments. For example, if the core data was collected from five countries, with five different minimum metal AP blast mines, such data could enable an overall risk assessment for the excavation of AP blast mines.

There are other limitations. One was the absence of differential GPS. This is essential for recording the position of each mine in relation to others. On this basis land release decision making can be made. Also, in order for the CDM to develop useable data to monitor the effectiveness of detectors in the field, a means of accurately and consistently assessing soil types will be required. This is a further area of development in the application of the model. One improvement that has been made since the end of the trial period is the adoption of individual barcodes for each mine in order to further strengthen data integrity.

## 6 Potential future developments

The field trial for the CDM will continue and further development may be made. One key problem for field operations are mines missing from where they are anticipated within the demining pattern. At the time of the trial in this scenario deminers were required to fully excavate 1.1 m by 1.1-m surface area box around the anticipated position of the mine down to 20 cm, and then check with the detector down to 33 cm in depth [24]. This slows demining progress and APOPO would wish to explore alternatives. One alternative would be to use two detectors on missed mine areas with the aim that this would be sufficient to prove there is nothing in the 1.1-m squared box down to 33 cm. In order to achieve acceptance of this technique as credible, an evidence base would be required. The CDM can assist in developing evidence for or against this technique by checking every signal with at least two detectors, and recording the respective signals where a mine is found in the CDM. At present the serial number and settings (black endcap) for the Minelab F3 detector that signalled on each mine is recorded, along with the depth of the mine. If the equivalent data was recorded for a second detector of a different model, with a further field for the equivalence of the signal, this could provide the evidence base on which a potential change to the missing mine drill could be made, and time and resources saved. For example, if it could be shown that for thousands of documented instances the second detector could pick up all R2M2 signals, including signals down to 33 cm, this could be sufficient to enable a change in national missing mine drills. Such a trial would require independent verification to ensure the evidence is reliable.

Other future developments may include applying the CDM to not only to VOEDs, such as anti-personnel mines, but also in a limited way in explosive remnants of war found in the course of debris removal from former combat zones. If the device, process, location and image data can be collected for each item of explosive ordnance found in the rubble, the risk that explosive ordnance presents during rubble removal of millions of tonnes can better assessed. For example, in its most simple terms, if all the explosive ordnance found is unfuzed Abandoned Explosive Ordnance (AXO), this represents a lower risk than if fuzed Unexploded Ordnance (UXO) is found. UXO is more likely to detonate on receipt of a mechanical insult. The CDM may also be adapted to assisting surveillance of the potential pollution from items of UXO found in the field. The CDM can capture the detail of individual items of explosive ordnance, alongside soil samples from around and under the item. In this way greater context and potentially explanation for each soil sample can be recorded.

## 7 Conclusion

The trial of the CDM has enabled the development of a semi-quantitative risk assessment for the excavation of R2M2 anti-personnel mines. Other risk assessments, such as for missing mines, can also now have a semi quantitative basis by means of CDM data. It has also provided an enhanced overview of operations for demining managers that enables improved quality and overall operations management. Operations managers can know and understand their own operations with CDM data in a way not previously possible.

The trial has also shown that the vastly expanded collection of data in relation to an accountable individual mine is entirely practicable and does not impose a significant burden on deminers in the field or their team leaders responsible for completing the forms. The CDM is practical to implement, for example if the core CDM were adopted at scale across all manual demining operations, the sector could start to assess comparative risk at an organisational level, not just at a country level. It could also be a means for increased transparency for the mine action sector with each mine now an accountable unit that can be verified by donors funding operations.

Each mine removed from the ground may be seen as a data opportunity. The CDM trial in Zimbabwe with APOPO has taken this opportunity with 877 mines. The opportunity exists to do the same for all VOEDs found in the future. This not only places much of field risk management in demining on an evidential semi-quantitative basis, it can also illuminate operations for all involved. The opportunity is there for the sector should it wish to take it forward.

In summary, it is believed that the trial data recorded for this research is the most detailed demining data set ever collected. This work will enable not only enhanced risk management by means of semi-quantitative risk assessments, but also supports overall quality and operations management. Furthermore, the collection of this data has proven to be entirely practicable and not to interfere in productivity or efficiency of demining teams. Indeed, the CDM enables systematic measurement of efficiency that can inform attempts to improve it in the future.

## CRediT authorship contribution statement

**R. Evans:** Writing – original draft, project administration. **M. Bold:** Project administration, writing – review & editing. **L. Nelson:** Writing – review & editing. **T. Temple:** Writing – review & editing.

## Data availability statement

No new data were created or analysed during this study. Data sharing is not applicable to this article.

## Declaration of competing interest

The authors declare the following financial interests/personal relationships which may be considered as potential competing interests: Associate Editor Heliyon If there are other authors, they declare that they have no known competing financial interests or personal relationships that could have appeared to influence the work reported in this paper.
